# Sparse measures with swarm-based pliable hidden Markov model and deep learning for EEG classification

**DOI:** 10.3389/fncom.2022.1016516

**Published:** 2022-11-16

**Authors:** Sunil Kumar Prabhakar, Young-Gi Ju, Harikumar Rajaguru, Dong-Ok Won

**Affiliations:** ^1^Department of Artificial Intelligence Convergence, Hallym University, Chuncheon, South Korea; ^2^Department of Electronics and Communication Engineering, Bannari Amman Institute of Technology, Sathyamangalam, India

**Keywords:** EEG, sparse representation, hidden Markov model, swarm intelligence, deep learning

## Abstract

In comparison to other biomedical signals, electroencephalography (EEG) signals are quite complex in nature, so it requires a versatile model for feature extraction and classification. The structural information that prevails in the originally featured matrix is usually lost when dealing with standard feature extraction and conventional classification techniques. The main intention of this work is to propose a very novel and versatile approach for EEG signal modeling and classification. In this work, a sparse representation model along with the analysis of sparseness measures is done initially for the EEG signals and then a novel convergence of utilizing these sparse representation measures with Swarm Intelligence (SI) techniques based Hidden Markov Model (HMM) is utilized for the classification. The SI techniques utilized to compute the hidden states of the HMM are Particle Swarm Optimization (PSO), Differential Evolution (DE), Whale Optimization Algorithm (WOA), and Backtracking Search Algorithm (BSA), thereby making the HMM more pliable. Later, a deep learning methodology with the help of Convolutional Neural Network (CNN) was also developed with it and the results are compared to the standard pattern recognition classifiers. To validate the efficacy of the proposed methodology, a comprehensive experimental analysis is done over publicly available EEG datasets. The method is supported by strong statistical tests and theoretical analysis and results show that when sparse representation is implemented with deep learning, the highest classification accuracy of 98.94% is obtained and when sparse representation is implemented with SI-based HMM method, a high classification accuracy of 95.70% is obtained.

## Introduction

In order to capture the activity of the brain, electroencephalography (EEG) signals are used which are nothing but the electrophysiological recordings of electrical potentials across the cortical regions of the brain (Lee et al., 2018). The spontaneous electrical activity of the brain in a very short span of time is thus measured by EEG. For analyzing various neurological-related disorders, such as coma, anesthesia, epilepsy, sleep disorders, schizophrenia, alcoholism, brain death, and encephalopathies, EEGs are widely used (Chen et al., 2016). During earlier times, the analysis was based only on visual inspection and interpretation that lead to more errors and also it required extensive training by the clinicians. With the advent of both specialized data acquisition devices and computer technology, identifying abnormalities have been incorporated very successfully (Lee et al., 2019). As EEG signals are extremely complex when compared to other biomedical signals, specialized and versatile feature extraction and selection methods incorporated with classification techniques have to be utilized. In this process, the selection of the most important features is highly useful and significant as it depicts the subsets of discriminant patterns (Won et al., 2018). Once that is achieved, the classification accuracy can be enhanced, the curse of the dimensionality problem can be alleviated, and thus the generalization capability of the system enhances gradually (Lee et al., 2015). This kind of methodology is adopted in a typical biomedical signal processing work and in this work since epilepsy classification and schizophrenia classification from EEG signals are discussed, a few important and relevant past literature in recent years is discussed as follows.

Plenty of articles are available online for epilepsy classification as it is a well-established research field nearly for the past two decades, and only a few articles are available online for schizophrenia classification as it has triggered interest among researchers very recently. A comprehensive review of the different machine learning techniques for epilepsy classification was reported in Sharmila and Geethanjali (2019), and the latest deep learning techniques utilized for epilepsy classification from EEG signals were analyzed thoroughly in Shoeibi et al. (2007). These two survey articles published in 2019 and 2020 review all the past works, working methodologies, statistical feature analysis techniques used, and datasets analyzed along with the comparison of classification accuracies obtained by every method, thereby easing the work of other researchers to not reproduce the past literature over and over again. However, some prominent ideas reported in high-quality literature during 2020 and 2021 for both epilepsy classification and schizophrenia classification are discussed as follows. An automated classification of epilepsy from EEG signals based on spectrogram and CNN was utilized in Mandhouj et al. (2021) reporting a classification accuracy of 98.25%. By means of integrating the property of convolutions with Support Vector Machine (SVM), a hybrid methodology called as Convolution SVM (C-SVM) was developed in Xin et al. (2021) reporting a classification accuracy of 99.56%. The optimal wavelet features were selected and combined with Long-Short Term Memory (LSTM) for epilepsy classification from EEG signals reporting a classification accuracy of 99% (Aliyu and Lim, 2021). Based on Jacobi polynomial transforms and Least Squares SVM, the classification of epilepsy was done in Nkengfack et al. (2021), reporting a classification accuracy ranging from 88.75 to 100%. The concept of synchrosqueezing transforms was utilized with standard machine learning techniques reporting a classification accuracy of 95.1% (Cura and Akan, 2021). A deep neural network model based on CNN is utilized for the analysis of robust detection of epileptic seizures from EEG signals reporting classification accuracy in the ranges of 97.63–99.52% (Zhao et al., 2020). A deep CNN with 10-fold cross-validation methodology was also implemented for epilepsy classification reporting a high classification accuracy of 98.67% (Abiyev et al., 2020). Other works discussed in this study are for the sake of comparing the proposed results with the previous works as the results implemented in this work were done with those same datasets. Different approaches for epilepsy classification included the usage of genetic programming (Bhardwaj et al., 2016), complex-valued classifiers (Peker et al., 2016), Empirical Mode Decomposition (EMD) based supervised learning (Riaz et al., 2016), weighted complex networks analysis (Diykh et al., 2017), Support Vector Machine (SVM) based automated seizure analysis (Zhang and Chen, 2017), and Recurrent Elman neural network classifier (Raghu et al., 2017) are some of the prominent works in this field of epilepsy classification. Recent approaches utilized for epilepsy classification in the past three years involve the usage of deep learning by means of proposing a Pyramidal 1D-CNN (Ullah et al., 2018), Continuous Wavelet Transforms with CNN (Turk and Ozerdem, 2019), and a simple normalization with a 1D-CNN (Zhao et al., 2020). Entropy-based analysis included the usage of fuzzy entropy and distribution entropy for seizure classification (Li et al., 2018) and a Fourier–Bessel series expansion-based rhythms splitting depending on weighted multiscale Renyi Permutation Entropy for epilepsy classification (Gupta and Ram, 2019). Other approaches incorporated are the usage of orthogonal wavelet filtering methodology (Sharma et al., 2018), matrix determinant approach (Raghu et al., 2019), and alpha band statistical feature-based detection of epileptic seizures (Sameer and Gupta, 2020). All these recent previous literature works are done on different epileptic datasets depending on their classification problem requirement, with some researchers focusing only on a single epileptic dataset while other authors concentrate on multiple epileptic datasets. When it comes to schizophrenia classification, many research results reported in high-quality literature are not available, and therefore, a selected few ones are presented in this study to get a clear understanding. An interesting methodology of schizophrenia classification from EEG was reported in Prabhakar et al. (2020a), where using three different features such as isometric mapping features, nonlinear regression features, and expectation maximization based principal component features was optimized using nature-inspired algorithms and classified with Modest Adaboost classifier reporting a classification accuracy of 98.77%. Another methodology for schizophrenia classification from EEG utilizes the standard statistical features such as Hurst exponent, Sample Entropy, and Detrend Fluctuation Analysis (DFA) with four kinds of optimization techniques, and finally, when it was classified with SVM, a classification accuracy of 92.17% was reported (Prabhakar et al., 2020b). Finally, a deep learning methodology was also involved using a 11-layer CNN for schizophrenia classification in Oh et al. (2019) and they reported a classification accuracy of 81.26% for subjects-based testing and 98.51% for non-subject-based testing. All the works proposed in the literature have its own merits and demerits, and consistent improvement is being made by researchers constantly with the usage of new ideas and methods so that the performance is improved.

In recent years, the sparse representation of the signals has received huge attention (Schoellkopf et al., 2007). The most compact signal representation is solved by a sparse theory that models a signal in the context of the linear combination of atoms in an overcomplete dictionary. The signals when represented in both multi-scale and multi-orientation aspects such as contourlet, ridgelet, wavelet, and curvelet transforms play an important role in the progress of research on sparse representation. For efficient signal modeling, a better performance is provided by sparse representation when compared to techniques based on direct time domain processing. On three different aspects of the sparse representation, the focus of sparse representation research is usually concerned, (a) pursuit techniques for solving the optimization problems, (b) dictionary design techniques, and (c) application of sparse representation for various tasks (Schoellkopf et al., 2007). The primary objective in the standard theory of sparse representation is to mitigate the signal reconstruction errors utilizing a very few number of atoms. In literature too, the application of sparse representation for modeling and classification has been well explored. Sparse representation for signal classification (Schoellkopf et al., 2007) and EEG classification based on sparse representation with deep learning (Gao et al., 2018) are the two most important applications of sparse concepts in biomedical signal processing. A widely utilized generative model is HMM which usually deals with sequential data and it assumes that based on a specific state of hidden Markov chain, the conditioning of every observation is done (Rezek and Roberts, 2002). It is a very famous probabilistic model where the general assumption is that a signal is generated by means of the utilization of a double-embedded stochastic process. For analyzing sequential data, HMMs are highly useful as the dynamics of the signal is encoded by a discrete-time hidden state process which projects as a Markov chain. At each instant of time, the appearance of the signal is encoded by an observation process and it is conditioned on the present state. For biomedical signal analysis especially the EEG, HMMs are highly useful and a few applications utilizing them for various aspects of EEG signal processing are ensemble HMM for analyzing EEG, parallel HMM to classify the multichannel EEG patterns, detection of various brain diseases from EEG signals using HMM and an obstructive sleep apnea detection approach using a discriminative HMM from EEG (Eberhart et al., 2001). Swarm Intelligence combined with HMMs serves as a good combination and has been successfully implemented in our work.

The main contributions of this work are as follows:

a) An efficient sparse representation model with sparseness measures analysis with the usage of Analysis Dictionary Learning Algorithm (ALDA) for the biosignal datasets has been implemented and no literature in the past have reported it for epileptic EEG signal classification and schizophrenia EEG signals classification.
b) A swarm intelligence–based pliable HMM has been developed and incorporated in this study and it is the first of its kind to do after the sparse representation analysis is done, as no literature in the past has proceeded in this methodology.
c) The sparse-modeled features are also classified with deep learning methodology using CNN and other traditional pattern recognition techniques for providing a comprehensive analysis.
d) Overall, the amalgamation of these techniques in this proposed kind of methodology is totally new and it can be successfully implemented in other biosignal processing datasets, imaging applications, speech signal processing, financial risk level assessment classification, biometrics, etc.

**FIGURE 1:**
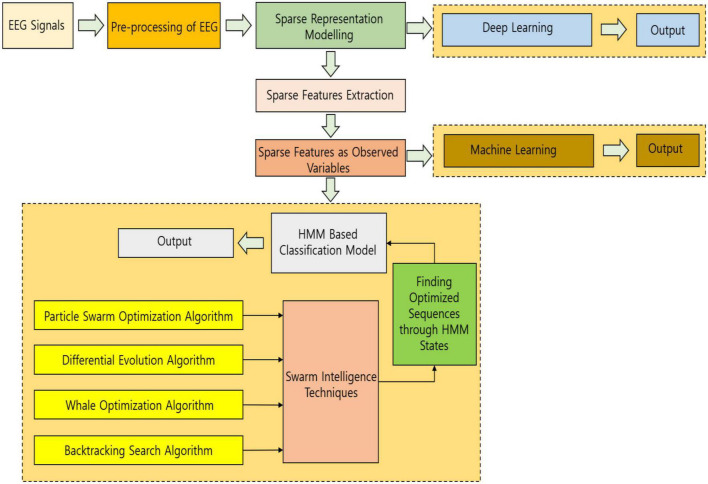
A simplified block diagram of the work for easy understanding.

In this work, sparse modeling is implemented with HMM ideology controlled by SI techniques and it is the first of its kind to adopt this methodology for biosignal processing datasets, making the system more versatile and adaptable. The organization of the work is as follows. The simplified block diagram of the work for an easy understanding is projected in Figure 1. Section “Sparse representation model” explains the sparse representation model of the EEG signals. Section “Hidden Markov model analysis” explains the modeling of HMM followed by the usage of swarm intelligence techniques and the incorporation of the deep learning methodology is explained in section “Deep learning–based methodology.” The results and discussion with experimentation and dataset details are projected in section “Results and discussion” and conclusion in section “Conclusion and future work.”

## Sparse representation model

The notations utilized in analyzing the sparse representation concept are explained as follows. An upper case alphabet *Z* denotes a matrix and lower case letter *z*_*ij*_ expresses the *ij^th^* entry of *Z*. A vector is defined by the lower-case letter, such as *z*. The *j^th^* entry of *z* is expressed as *z*_*j*_. The *i^th^* row and *j^th^* column of a particular matrix *Z* is defined by the matrix slices *Z*_*i*:_ and *Z*_:*j*_, respectively. For a matrix *Z*, the Frobenius norm is expressed as ||*Z*||_*F*_ = (Σ_*i*,*j*_|*z*_*ij*_|^2^)^1/2^. To indicate the determinant value of a specific matrix, det⁡(•) is utilized.

### Sparse signal representation

With the help of sparse representation, the observed signals are decomposed into a unique product of a dictionary matrix which will have the signal base and along with it a sparse coefficient matrix will also be present (Schoellkopf et al., 2007). A synthesis model and analysis model are the two various structures of the sparse representation model. The firstly initiated sparse model is the synthesis model and it is very widely utilized. Assuming that the modeling of signals to be done as *Z* ∈ ℜ^*p*×*N*^, where the signal dimensionality is represented as *p* and the total number of measurements are represented as *N*. The signals could be expressed in the synthesis sparse model as


(1) Z=D⁢G

(2) Z≈D⁢G

(3) $$\mathrm{such}\,\mathrm{that}\,{||\text{Z-DG}||}_{F}^{2}\leq \varepsilon ,$$

where *D* ∈ ℜ^*p*×*n*^ is considered as a dictionary, *G* ∈ ℜ^*n*×*N*^ denotes a representation co-efficient matrix, and a very small residual factor is given by ε ≥ 0. The number of bases is represented by ′*n*′ and it is termed as dictionary atoms. To obtain the sparse representation of the signals, it is assumed that from the dictionary matrix *D*, the representation matrix *G* is sparse in nature (i.e., numerous zero entities). From equations (1) and (2), it implies that the representation of every signal is done as a linear combination of a few atoms.

The choice of solution for the dictionary is the most important key issue of the sparse representation which the discovered signals are utilized to decompose. The famous choices are either a pre-defined dictionary such as wavelets, Discrete Fourier Transform (DFT), and Discrete Cosine Transform (DCT) or a learned dictionary which results to match the contents of the signals in a better manner (Schoellkopf et al., 2007). In real-world applications, a better performance is exhibited by the learned dictionary when compared to the pre-defined dictionaries. The analysis model is a simple and interesting twin of the synthesis model and it should be considered important. Supposing that there is a matrix Ω ∈ ℜ^*n*×*p*^ that gives a sparse coefficient matrix _*G*_ by means of being multiplied by the signal matrix *G* = Ω*Z*.

For the error function ||*G* − Ω*Z*||_*F*_, there is a minimization problem and the equation *G* = Ω*Z* can be utilized as a solution to it. The standardized optimization methods can be very well deployed in this study as the error function is convex. To perform optimization in the analysis model is very easy as the error function present in the synthesis model is non-convex in nature. Now the analysis dictionary is represented as Ω ∈ ℜ^*n*×*p*^. In the analysis dictionary Ω, the atoms are considered as its rows rather than the consideration of atoms as columns in the synthesis dictionary *D*. In order to assemble a sparse result, the dictionary analyses the signal and so the term “analysis” is used. To clearly distinguish and stress the importance between analysis and synthesis models, a co-sparsity has been utilized (Gao et al., 2018), which helps in counting the number of zero-valued elements of Ω*Z*, which is nothing but the zero elements co-produced by Ω and *Z*. Therefore, the cosparse model can also be used instead of sparse model, and cosparse dictionary can also be used instead of analysis dictionary.

Now analysis sparse model is examined more carefully. The analysis model represented for one signal *z* ∈ ℜ*^p^*, which is a column in the signal matrix *Z* is now indicated utilizing an acceptable analysis dictionary Ω ∈ ℜ^*n*×*p*^. The *i^th^* row termed as the *i^th^* atom in Ω is specified by *q*_*i*_. Now the analysis representation vectors *g* = Ω*z* should be made sparse and it is done by means of introducing a sparse measure *M*(*g*), so that the behavior becomes negatively influenced by the sparsity nature of *g* and therefore by mitigating *M*(*g*), it gives the sparsest solution represented as


(4) $$\begin{array}{c} \Omega =\underset{\Omega }{\arg\min}M\,\left(g\right)\\ s.t\,\begin{array}{cc} & g=\Omega \,z \end{array} \end{array}$$

By utilizing *l*_0_ norm thoroughly by means of setting *M*(*g*) = ||*g*||_0_, the sparsest solution is obtained. Such a constraint leads to often NP hard problem and the optimization problem becomes combinatorial. To have easier optimization problems, the other sparsity measures such as the *l*_1_ norm are utilized. It is also known that utilizing *l*_1_ norm can lead to the solution becoming too sparse as it often over-penalizes large elements.

### Sparseness measures analysis

For estimation and appraisal of the sparseness of a vector, the *l*_*p*_ norms are highly useful and are popularly used, where *p* = 0,1, or 2. An NP-hard problem is often yielded by the *l*_0_ norm, and therefore, *l*_1_ norm has its convex evaluation utilized often (Gao et al., 2018). For a vector *g*, the *l_1_* -norm is expressed to be the total sum of the absolute values of *g*; i.e., ||*g*||_1_ = Σ_*i*_|*g*_*i*_|.

For non-negative vectors, *g* ∈ ℜ_+_, the *l*_1_ -norm of *g* is expressed as ||*g*||_1_ = Σ_*i*_*g*_*i*_. The *l*_1_ -norm is usually smooth and differentiable for non-negative vectors and therefore such gradient techniques are utilized in optimization. The introduction of *l*_2_ -norm with non-negative matrix factorization is sometimes considered as its yields sparse solutions. The results with *l*_0_ -norm or *l*_1_ -norm are more sparser than the results with *l*_2_ norm. The instantaneous sparsity nature of only one signal can be expressed by the sparsity measures mentioned above and are generally not utilized for covering and evaluating the sparsity across various sources of measurement.

For non-negative sources, a determinant type of sparsity measure is employed to express the joint sparseness. The sparseness of non-negative matrices can be explicitly measured by the determinant-sparse type and measures as it has various good qualities. The determinant value of a non-negative matrix is well bounded if the normalization of a non-negative matrix is done, thereby interpolating its value between two extremes 0 and 1, and thus enhancing the sparsity. Supposing if the non-negative matrix *Y* is non-sparse, then the determinant of *YY*^*T*^, det⁡(*YY*^*T*^) addresses toward 1. If all the entries of *YY*^*T*^ are similar, then the determinant value acts in a manner such that 0 ≤ det (*YY*^*T*^) ≤ 1, where det (*YY*^*T*^) = 0. The following two conditions are fully complacent at the time when det (*YY*^*T*^) = 1 and are mentioned as follows:

(i) For all *i* ∈ {1, 2, …, *p*}, only a single element in *y*_*i*_ is non-zero
(ii) For all *i*, *j* ∈ {1, 2, …, *p*}, and *i* ≠ *j*, *y*_*i*_, and *y*_*j*_ are orthogonal in nature, $y_{i}^{T}\,y_{j}=0$

Thus, in the cost function, the determinant measure can be utilized. If the determinant measure has a larger value, then the matrix is more sparse. Therefore, with these determinant constraints, the sparse coding problem can be now modeled as an optimization problem and represented as


(5) $$\max_{y}\det\left(Y\,Y^{T}\right)=\min_{y}-\det\left(Y\,Y^{T}\right)$$

### Formulation of sparse representation problem

The analysis sparse representation problem description is explained as follows. It is assumed that the observed signal vector $t\in {ℜ}_{+}^{p}$ is present and it is a noisy aspect of a signal $z\in {ℜ}_{+}^{p}$. Therefore, *t* = *z* + *v*, where *v* denotes additive positive white Gaussian noise. With the help of an analysis dictionary Ω ∈ ℜ^*n*×*p*^, every row which explains 1 × *p* analysis atom is considered so that *z* satisfies ||Ω*z*||_0_ = *p* − *s*, where *s* expresses the cosparsity of the signal which is matched to be the total number of zero elements. To define the signals with every column as one signal, a matrix *Z* is utilized so that the signals matrix can be extended. In this study, the sparse measure is analyzed as *M*(.). The noise in the measured signals is considered, and therefore, for analyzing dictionary learning, an optimization task is formulated as


(6) $$\min_{\Omega ,Z}M\,\left(\Omega \,Z\right)$$

such that ${||\text{T-Z}||}_{F}^{2}\leq \sigma$.

The noise level parameter is denoted by σ, the sparse regularization is expressed as *M*. With the help of penalty multipliers, a regularized version of the above equation can be done. In such a case, *X* is considered as an approximation of Ω*Z*, which tends to make the learning fast and easy. By means of thresholding the sparsity measure on *X* and the product of Ω*Z*, the analysis sparse coding is obtained.

The analysis sparse representation is expressed as


(7) $$\min_{\Omega ,Z,X}M\,\left(X\right)+\lambda \,{||\text{T-Z}||}_{F}^{2}+\beta \,{||\Omega \,\text{Z-X}||}_{F}^{2}$$

such that ||*q*_*i*_||_2_ = 1, ∀_*i*_, where the representation coefficient matrix is denoted by *X* ∈ ℜ^*n*×*N*^. Now the representation matrix *X* is considered as sparse. The λ and β in (7) are estimated with the help of the famous Lower Upper (LU) decomposition technique. To remove the scale ambiguity, a normalization constraint is introduced (∀_*i*_||*q*_*i*_||_2_ = 1). The analysis dictionary learning procedure is summarized in Algorithm 1.

**Algorithm 1:** Analysis dictionary learning algorithm (ADLA).

Initialization: Ω_0_, *X*_0_, *Z*_0_ = *T*, *i* = 0 While convergence is not achieved do $\Omega_{i+1}=\min_{\Omega }\,{||\Omega \,Z-X||}_{F}^{2}\,s.t.\forall_{i}{||q_{i}||}_{2}=1$ $X_{i+1}=\min_{X}M\,\left(X\right)+\beta \,{||\Omega \,Z-X||}_{F}^{2}$ $Z_{i+1}=\min_{Z}\lambda \,{||T-Z||}_{F}^{2}+\beta \,{||\Omega \,Z-X||}_{F}^{2}$ *i* = *i* + 1

For the sparse represented EEG signal, the statistical feature parameters such as mean, variance, skewness, kurtosis, sample entropy, approximate entropy, Shannon entropy, Hurst exponent, Largest Lyapunov Exponent, Fractal Dimension, Recurrence Quantification Analysis, Higher Order Cumulants, Lempel Ziv Complexity, Kolmogorov Complexity, and Hjorth exponent are computed. Table 1 shows the average statistical feature parameter values for sparse represented EEG data signals. It is noted from Table 1 that low values of mean, variance, and skewness are observed among the Bonn dataset (Andrzejak et al., 2001) (normal, inter-ictal, and ictal categories) and schizophrenia dataset, while the kurtosis parameter reached a high value in the Bonn dataset and schizophrenia dataset (Olejarczyk and Jernajczyk, 2017). Bonn dataset does not differentiate among the entropy features, but in the case of schizophrenia dataset, there exists a difference in the entropy features. All the statistical parameters for the features such as Hurst Exponent, Largest Lyapunov Exponent, Fractal Dimension, Recurrence Quantification Analysis, Higher Order Cumulants, Lempel Ziv Complexity, Kolmogorov Complexity, and Hjorth Exponent show the nonlinear behavior and it has very close values among the group of datasets. This justification indicates that the sparse represented data should be further processed through the HMM with bio-inspired learning algorithms.

**TABLE 1:** Average statistical feature parameters for sparse represented EEG dataset signals.

Sl. No.	Statistical parameters	Bonn EEG dataset			Schizophrenia dataset	
		A	C	E	Schizophrenia	Normal
1	Mean	0.10049	0.300822	0.850364	0.971108	0.189563
2	Variance	0.001707	0.001091	0.000505	3.9E-05	5.31E-05
3	Skewness	0.561381	1.5384	–0.69973	0.596389	1.523915
4	Kurtosis	64.37504	48.20223	29.77208	59.32448	77.81783
5	Sample entropy	11.7308	11.5397	11.3726	6.8751	10.289
6	Approximate entropy	1.986	1.648	1.461	1.7916	2.041
7	Shannon entropy	10.87	6.69	5.421	5.832	11.67
8	Hurst exponent	0.734	0.582	0.348	0.231	0.831
9	Largest Lyapunov	0.839	0.2311	0.469	0.415	0.942
10	Fractal dimension	0.2769	0.281	0.286	0.341	0.242
11	Recurrence quantification	0.1208	0.1176	0.2177	0.2307	0.098
12	Higher order cumulants	0.2495	0.482	0.725	0.774	0.2116
13	Lempel–Ziv complexity	341.33	334.9	326.58	406.91	312.21
14	Kolmogorov complexity	11.039	9.873	9.5684	7.8002	7.6749
15	Hjorth exponent	1.528	1.7153	1.6887	1.6002	1.726

## Hidden Markov model analysis

To express a Markov process with unknown parameters, HMM is often used (Rezek and Roberts, 2002). Through observable parameters, it is hectic to understand the implicit parameter of the process, and so it is utilized to proceed with further in-depth analysis. Two discrete-time stochastic processes that are related to each other are described by HMM. Hidden state variables are applicable to the first process and denoted as (*V*_1_, *V*_2_, …, *V*_*n*_), which emits the observed variables with various probability factors. The second process is applicable and related to observed variables (*w*_1_, *w*_2_, …, *w*_*n*_). The transition probability and the emission probability are the two main parameters of HMM.

Transition Probability: *P* (*V*_*l*_ = *v*_*p*_|*V*_*l*−1_ = *v*_*m*_) It implies that the current state depends on the previous state *v*_*m*_.

Emission Probability: *P* (*w*_*l*_|*V*_*l*_ = *v*_*p*_) The current state *v*_*p*_ is used to release the observation symbol. In our model, for every extracted sparse signal feature *f*_*i*_, an HMM λ^(*f*_*i*_)^ is built. The observed variables are nothing but the sparse representation features extracted from the signal ′*s*′, while every hidden state $V_{l}^{\left(f_{i}\right)}$ of λ^(*f*_*i*_)^ is assured as a state related to the feature *w*_*l*_. If the sparse representation *S* obtains a very high probability for the model λ^(*f*_*i*_)^, it implies that *S* is related to the sparse signal feature *f*_*i*_.

### Consideration of sparse features as observed variables

The features extracted from the sparse signal model are termed as sparse features, and these are considered as observed variables. Under this domain, category-based extraction and global-based extraction are the two main categories of feature extraction techniques. As global-based extraction methods cannot be utilized to differentiate the various sparse features so well, in our work we adopted category-based feature extraction techniques.

Considering a set of *n* feature extraction techniques {*F*_1_, *F*_2_, …, *F*_*n*_}, a sparse representation *S* is divided into *n* terms (*k*_1_, *k*_2_, .., *k*_*n*_). Assuming *z*_*jl*_ is the *l^th^* feature which is extracted by the method *F*_*l*_. For computing the sparse representation feature vector, an intermediate *h* × *n* matrix of term-level feature is utilized. For every sparse signal feature *f*_*i*_, the sparse representation feature vector of the signal *S* is represented as follows:


(8) $$\left[\begin{array}{c} k_{1}\\ k_{2}\\ :\\ k_{h} \end{array}\right]\to \left[\begin{array}{cccc} z_{11} & z_{12} & .. & z_{1\,n}\\ z_{21} & z_{22} & .. & z_{2\,n}\\ : & : & : & :\\ z_{h\,1} & z_{h\,2} & .. & z_{h\,n} \end{array}\right]\to {\left[w_{1},w_{2},..,w_{n}\right]}^{\left(f_{i}\right)}$$

where $w_{l}=\sum_{j=1}^{h}z_{j\,h}/h,\left(1\leq l\leq n\right)$.

Over all the signal features,*w*_l_ is a mean value of _*l*^*th*^_ features over all the extracted signal features.

### Development of hidden Markov model-based signal classification model

A value is supposed to be emitted by each hidden state and so the sequence of values is generated by the whole model that constitutes and manages the sparse representation feature vector. The representation of the best signal category is done by a set of values and it is considered to be as a state in our work. Between the sparse representation and the HMM states, there is a one-to-one mapping that requires the transition of hidden states to be in a stationary mode and the states to indicate the start level *v*_1_. During the working of the classifier, the features of test sparse representations being drawn closer to the signal are done by the transition probability and are expressed as follows:


(9) $$P\left(V_{l}=v_{p}|V_{l-1}=v_{m}\right)=\left\{ \begin{array}{c} 1,\left(p=m+1\right)\\ 0,\left(p\neq m+1\right) \end{array}\right.$$

With the help of known state *V*_*l*_, the feature *w*_*l*_ and the training data, the emission probability *P* (*w*_*l*_/*V*_*l*_)is calculated. The HMM model λ^(*f*_*i*_)^ for every feature *f*_*i*_ is expressed in Algorithm 2 as follows:

**Algorithm 2:** Expression of every feature in the HMM Model.

The signal level feature vector is expressed as [*w*_1_, *w*_2_, …, *w*_*n*_]^(*f*_*i*_)^ Input: feature vector [*w*_1_, *w*_2_, …, *w*_*n*_]^(*f*_*i*_)^ Output: Probability *P*([*w*_1,_*w*_2_, …, *w*_*n*_]^(*f*_*i*_)^|λ^(*f*_*i*_)^) For *l* = 1 *to n do* Calculate *P* (*w*_*l*_/*V*_*l*_)^(*f*_*i*_)^ End for Calculate *P* ([*w*_1,_*w*_2_, …, *w*_*n*_]^(*f*_*i*_)^|λ^(*f*_*i*_)^) using forward algorithm.

For each of the signal features, the HMM concept is constructed and implemented. The calculation of the probabilities of the sparse representations on the signal feature models is done when a new sparse representation arrives. The sparse representation is labeled with the signal features whose model is highly related to the maximum probability.

To compute *P* (*w*_*l*_|*V*_*l*_)^(*f*_*i*_)^, a Jaccard similarity (*J*) is utilized which helps in testing the correlation between the value *w*_*l*_ and _*V*_*l*__.


(10) $$P\,{\left(w_{l}|V_{l}\right)}^{\left(f_{i}\right)}=J\,{\left(w_{l}|V_{l}\right)}^{\left(f_{i}\right)}=\frac{H_{11}}{H_{11}+H_{10}+H_{01}}$$

where *H*_11_ indicates the number of sparse representations contained with *w*_*l*_ and *V*_*l*_ in *f*_*i*_; *H*_01_ indicates the number of sparse representations which has only *w*_*l*_ in *f*_*i*_; *H*_10_ indicates the number of sparse representations which has only *V*_*l*_ in *f*_*i*_.

To assess whether the signal feature *f*_*i*_ relates to the observation *w*_*l*_ or the state *V*_*l*_, an associated factor is included and it is specified by δ_*l*_. The feature extracted from the training data be $w_{l}^{\prime }$ and the association is described as follows:


(11) $$\left|w_{l}^{\prime }-w_{l}\right|\leq \delta_{l}\,o\,r\,\left|w_{l}^{\prime }-V_{l}\right|\leq \delta_{l}$$

If the above inequalities are satisfied, then it is understood that the observation *w*_*l*_ or the state *V*_*l*_ is related to *f*_*i*_.

### Self-Pliable mechanism of hidden Markov model by swarm intelligence techniques- computation of parameters

It is very important to build a versatile HMM classifier, and it is significant to trace the optimized sequences of the HMM states. For the optimization of state parameters, various strategies are utilized by means of utilizing SI techniques. In this work, PSO, DE, WOA, and BSA are utilized. The main reasons for selecting these four SI techniques are because they are very easy and have a simple implementation with fast convergence and good computational efficiency. As HMM can adapt itself to the various optimization techniques, the HMM techniques can be called as self-pliable one.

#### Particle swarm optimization

A famous population-dependent stochastic optimization is PSO (Eberhart et al., 2001). The candidate solution of an HMM parameter is represented by every particle in PSO. Around the search space, the movement of the particles takes place. With the help of the local best-known position of a particle, the best-known positions are found. The best parameters can be iteratively found by this technique. PSO has the extreme power to achieve global optimization and it has a good application in our study. The mathematical expressions concerning it are as:


(12) $$v_{e}\,\left[\right]=W_{e}\times v_{e}\,\left[\right]+a\,c_{1}\times r\times \left(p\,b\,e\,s\,t\,\left[\right]-p\,r\,e\,s\,e\,n\,t\,p\,o\,s\,i\,t\,i\,o\,n\,\left[\right]\right)+a\,c_{2}\times R\times \left(g\,b\,e\,s\,t\,\left[\right]-p\,r\,e\,s\,e\,n\,t\,p\,o\,s\,i\,t\,i\,o\,n\,\left[\right]\right)$$

(13) $$p\,r\,e\,s\,e\,n\,t\,p\,o\,s\,i\,t\,i\,o\,n\,\left[\right]=p\,r\,e\,s\,e\,n\,t\,\left[\right]+v_{e}\,\left[\right]$$

[] specifies that its specific variable is a vector. In the range of [0,1], the variables *r* and *R* are represented.

The individual extremes are recorded by *pbest*[], and the global extremes are recorded by *gbest*[]. The inertia weight is represented by the constant *W*_*e*_. The acceleration constants are represented as *ac*_1_ and *ac*_2_.

Based on the previous velocity value and its corresponding distance to the best particle, the updates of the velocities of particles are done. The present [] particle’s position is updated by (13) based on the current velocity and the previous position value.

Parameter settings of PSO: To have various impacts on optimization performances, various PSO parameters are considered. The PSO parameters are selected on the following basis:

*V*_*max*_ : It is set by values of training data and it implements the searching space granularity.

*W*_*e*_ : It decides the motion inertia of the particles and the value is set as 0.5 in our experiment.

*ac*_1_, *ac*_2_ : indicates the accelerated weight so that it could propagate each particle to *pbest*[] and *gbest*[], the weights of both of them are set to 4 after a lot of trial and error basis.

To find the two types of parameters in HMM, a fitness function is used; (i.e.) the reduced associated factor δ_*l*_ and the $V_{l}^{\left(f_{i}\right)}$, which indicates the *l^th^* hidden state of the HMM λ^(*f*_*i*_)^.

The definition of fitness function is done as follows:


(14) $$f\,i\,t\,n\,e\,s\,s\,\left(\delta_{l},V_{l}^{\left(f_{1}\right)},\ldots ,V_{l}^{\left(f_{6}\right)}\right)=F_{1}-M\,e\,a\,s\,u\,r\,e,\left(1\leq l\leq n\right)$$

where *F*_1_ measure is one of the metrics used for classification accuracy. The exhaustive search is done for a total number of the involved parameters. The set of parameters is divided into *n* independent parts as training the whole parametric set is time-consuming by PSO. Depending on the fitness function, the parameters are thoroughly learned.

#### Differential evolution

It is a famous population-based approach that is widely used by everyone and is a promising global search technique and can be used well for HMM (Sarker et al., 2014). The candidate solution of an HMM parameter is represented by every evolution process in DE. Once the initial population is generated, then by looping mutation, selection, and crossover operations, the updation of the population is done. In the following four steps, the DE procedure is summarized as follows:

(A) Initialization: By utilizing random number distributions, the generations of an initial population are done. The *j^th^* dimension of the *i^th^* individual is initialized as


(15) $$z_{i,j}=B_{j}+r\,a\,n\,d\,{\left(0,1\right)}^{*}\,\left(U_{j}-L_{j}\right),i=1,2,\ldots ,S,j=1,2,\ldots ,D$$

where the population size is *S* and the dimension of individual is represented as *D*,

A random number in [0,1] range which is uniformly distributed is expressed by rand (0,1). The upper bound of the *j^th^* dimension is expressed as *U*_*j*_ and the lower bound of the *j^th^* dimension is expressed as *L*_*j*_, respectively.

(B) Mutation: The differential evolution enters the main loop after the initialization is done. A mutant individual *m*_*i*_ through mutation operators (*DE*/*rand*)/1 is generated by every target individual *z*_*i*_ in the population. The generated *m*_*i*_ is represented as


(16) $$m_{i}=z_{r\,1}+C^{*}\,\left(z_{r\,2}-z_{r\,3}\right),r_{1}\neq r_{2}\neq r_{3}\neq i$$

where *r*_1_, *r*_2_, and *r*_3_ are selected randomly from the present population. To scale the difference vector, *C* is utilized and is termed as the mutation control parameters.

The other generally used mutation operators for DE are expressed as follows:


(17) $$\begin{array}{c} \left(1\right)\,"\,D\,E/b\,e\,s\,t/1\,"\\ \,m_{i}=z_{b\,e\,s\,t}+C^{*}\,\left(z_{r\,1}-z_{r\,2}\right),r_{1}\neq r_{2}\neq i \end{array}$$

(18) $$\begin{array}{c} \left(2\right)\,"\,D\,E/r\,a\,n\,d/2\,"\\ \,m_{i}=z_{r\,1}+C^{*}\,\left(z_{r\,2}-z_{r\,3}\right)+C^{*}\,\left(z_{r\,4}-z_{r\,5}\right),\\ \,r_{1}\neq r_{2}\neq r_{3}\neq r_{4}\neq r_{5}\neq i \end{array}$$

(19) $$\begin{array}{c} \left(3\right)\,"\,D\,E/b\,e\,s\,t/2\,"\\ \,m_{i}=z_{b\,e\,s\,t}+C^{*}\,\left(z_{r\,1}-z_{r\,2}\right)+C^{*}\,\left(z_{r\,3}-z_{r\,4}\right),\\ \,r_{1}\neq r_{2}\neq r_{3}\neq r_{4}\neq i \end{array}$$

(20) $$\begin{array}{c} \left(4\right)\,“\,D\,E/C\,u\,r\,r\,e\,n\,t-t\,o-b\,e\,s\,t/1^{\prime \prime }\\ \,m_{i}=z_{i}+C^{*}\,\left(z_{r\,1}-z_{i}\right)+C^{*}\,\left(z_{r\,2}-z_{r\,3}\right),\\ \,r_{1}\neq r_{2}\neq r_{3}\neq i \end{array}$$

(21) $$\begin{array}{c} \left(5\right)\,“\,D\,E/r\,a\,n\,d-t\,o-b\,e\,s\,t/1^{\prime \prime }\\ \,m_{i}=z_{i}+C^{*}\,\left(z_{b\,e\,s\,t}-z_{i}\right)+C^{*}\,\left(z_{r\,1}-z_{r\,2}\right),\\ \,r_{1}\neq r_{2}\neq i \end{array}$$

*z*_*best*_ represents the individual with the best fitness function value.

However in this work, all the above-mentioned five combinations were utilized and upon analysis,(*DE*/*rand*)/1 was finally chosen and implemented as it was very convenient to set and alter the values after the initialization process is done.

(C) Crossover:

To generate a trial individual *t*_*i*_, a crossover operation which is binomial in nature is implemented to the target individual *z*_*i*_ and the mutant individual *m*_*i*_ as follows:


(22) ti,j={mi,j⁢i⁢f⁢r⁢a⁢n⁢d⁢(0,1)≤L⁢R⁢o⁢r⁢j=jr⁢a⁢n⁢dzi,j           ⁢o⁢t⁢h⁢e⁢r⁢w⁢i⁢s⁢e

where a randomly chosen integer in the range of [1, *D*] is expressed as *j*_*rand*_. The crossover control parameters are expressed as *CR* and it is in the range of *CR* ∈ [0,1].

(D) Selection:

Selection of the better one from the target individuals *z*_*i*_ and crossover individual *t*_*i*_ into the upcoming generations is important and so the greedy selection operator is utilized in this study. Based on the primary comparison of fitness values, this operation is performed, and it is computed as:


(23) $$z_{i}^{t+1}=\left\{ \begin{array}{c} t_{i},\begin{array}{cc} & i\,f\,\begin{array}{cc} f\,i\,t\,\left(t_{i}\right)<f\,i\,t\,\left(z_{i}\right) & \end{array} \end{array}\\ z_{i},\begin{array}{cccc} & & & \begin{array}{cc} & o\,t\,h\,e\,r\,w\,i\,s\,e \end{array} \end{array} \end{array}\right.$$

where the fitness function is denoted by *fit*.

#### Whale optimization algorithm

A famous swarm-based metaheuristic algorithm is WOA (Mirjalili and Lewis, 2016). The candidate solution of an HMM parameter is represented by every whale in WOA. The intelligent foraging behavior of hump back whales is mimicked in it, and this algorithm is influenced by bubble net hunting strategy (Mirjalili and Lewis, 2016). The main operators are included in WOA such as

(i) Simulation and searching the prey.
(ii) Encircling behavior of the prey.
(iii) Bubble net foraging behavior of the whales.

The exploration phase is nothing but searching for prey, and the exploitation phase is the encircling prey and spiral bubble net attacking method. For the two phases, the mathematical model is presented below.

(I) Initial stage: Exploitation stage

This includes the encircling prey phase/bubble net attacking method. Based on two mechanisms, the updation of their positions is done by the hump back whales during the exploitation phases such as shrinking with encircling mechanism and the spiral updation position. The former is called encircling prey, and the latter is called spiral bubble net attacking method. Using the following equations, the representation of the shrinking mechanism is done as follows:


(24) $$\vec{Z}\,\left(t+1\right)={\vec{Z}}^{{}^{*}}\,\left(t\right)-\vec{M}.\vec{D\,i\,s},$$

(25) $$\vec{D\,i\,s}=\left|\vec{N.}\,\vec{Z^{{}^{*}}}\,\left(t\right)-\vec{Z}\,\left(t\right)\right|$$

where the current iteration is represented by *t*.

The best solution of the position vector obtained so far is represented by $\overset{\to *}{Z}\left(t\right)$, and the position vector is indicated as $\vec{Z}\,\left(t\right)$.

The coefficient vectors are denoted as _$\vec{M}$_ and _$\vec{N}$_, and it is calculated as follows:


(26) $$\begin{array}{c} \vec{M}=2\,\vec{m}.\vec{r}-\vec{m}\\ \vec{N}=2.\vec{r} \end{array}$$

In both the phases, over the period of iterations, $\vec{m}$ is linearly decreased from 2 to 0. Here, $\vec{r}$ represents a random vector in the range of [0.1]. Using the following equation, the mathematical representation of the spiral updating position is expressed as follows:


(27) $$\vec{Z}\,\left(t+1\right)=\vec{D\,i\,s^{\prime }}.e^{c\,q}.\cos\left(2\,\pi \,l\right)+\vec{Z^{{}^{*}}}\,\left(t\right)$$

(28) $$\vec{D\,i\,s^{\prime }}=\left|\vec{Z^{{}^{*}}}\,\left(t\right)-\vec{Z}\,\left(t\right)\right|$$

The distance of the *x*^*th*^ humpback whale to the best solution derived is represented by $\vec{D\,i\,s^{\prime }}$.

The logarithmic spiral shape is defined by a constant *c* and the random number in the range of [–1,1] and is expressed by *q*. The element-by-element multiplication is given by (⋅). The mechanism exhibited by whale when catching a prey such as shrinking encircling mechanism and spiral updating positions are accomplished at the same time. The assumption is that a probability of 50% is chosen between them so that this behavior could be initiated. This mathematical modeling is expressed as follows:


(29) $$\vec{Z}\,\left(t+1\right)=\left\{ \begin{array}{c} \vec{Z^{{}^{*}}}\,\left(t\right)-\vec{M}.\vec{D\,i\,s}\,\begin{array}{cc} & i\,f\,\begin{array}{cc} & k<0.5 \end{array} \end{array}\\ \vec{D\,i\,s^{\prime }}.e^{c\,q}.\cos\left(2\,\pi \,q\right)+\vec{Z^{{}^{*}}}\,\left(t\right)\,\begin{array}{cc} & i\,f\,\begin{array}{cc} & k\geq 0.5 \end{array} \end{array} \end{array}\right.$$

where the random number *k* is in the range of [0,1].

(II) Prey Searching Phase (Exploration Phase):

In order to increase the exploration capability of WOA, based on randomly selected whale, the position of the whale is updated instead of utilizing the best whale food in the process. To force or to propagate away from a whale and to move very far from the best-known whale, a coefficient vector *M* with random values substantially greater than 1 or less than -1 is utilized.

Mathematically, it is expressed as


(30) $$\vec{Z}\,\left(t+1\right)={\vec{Z}}_{r\,a\,n\,d}\,\left(t\right)-\vec{M.}\,\vec{D\,i\,s}$$

(31) $$\vec{D\,i\,s}=\left|\vec{N.}\,{\vec{Z}}_{r\,a\,n\,d}\,\left(t\right)-\vec{Z}\,\left(t\right)\right|$$

where a random position vector selected from the current population is expressed as ${\vec{Z}}_{r\,a\,n\,d}$.

#### Backtracking search optimization algorithm

A famous population-based metaheuristic algorithm is BSA (Beek, 2006). The candidate solution of an HMM parameter is represented by every search in backtracking mechanism of BSA. By means of implementing mutation, crossover, and selection of population, this algorithm achieves the optimization purpose similar to other meta-heuristic algorithms. It has the unique quality to remember historical populations and therefore by completely mining the historical information, previous generations can be benefitted. Five steps are present in the original BSA, namely, (i) initialization (ii) Selection Phase I (iii) Mutation (iv) Crossover, and (v) Selection Phase II. The explanation for the 5 steps is as follows:

Step 1: Initialization:

At the outset, with the following formula, the population *A* and the historical population *oldA* is initialized by BSA, respectively.


(32) $$\begin{array}{c} A_{i,j}∼W\,\left(l\,o\,w_{j},u\,p_{j}\right)\\ O\,l\,d\,A_{i,j}∼W\,\left(l\,o\,w_{j},u\,p_{j}\right) \end{array}$$

where *i* = 1, 2, …, *S*, *j* = 1, 2, …, *D*.

The population size is represented by *S*, and the population dimension is represented by *D*, respectively. The uniform distribution is denoted by *W*. The lower boundaries of variables are denoted as *low*_*j*_, and the upper boundaries of variable are denoted as *upp*_*j*_.

Step 2: Selection Phase I:

Based on equation (32), the updation of the historical population *oldA* is done. Then there is a random change in the locations of individuals in *oldA* as projected in equation (33):


(33) $$i\,f\,\begin{array}{cc} p<q\left(p,q∼W\left(0,1\right)\right) & ,\begin{array}{cc} t\,h\,e\,n & o\,l\,d\,A=A \end{array} \end{array}$$

(34) o⁢l⁢d⁢A=p⁢e⁢r⁢m⁢u⁢t⁢i⁢n⁢g⁢(o⁢l⁢d⁢A)

where a random permutation of the integers from 1 to *N*is done by permuting (⋅) operations.

Step: 3 Mutation Process

The initial trial population is generated by the mutation operator of BSA so that there is complete control of the documented and authentic information along with the current information. The expression of mutual operation is expressed as:


(35) $$M_{i,j}=A_{i,j}+C^{*}\,\left(o\,l\,d\,A_{i,j}-A_{i,j}\right)$$

where the control parameters are denoted by *C*, and the value of *C* is chosen to be 5 in our experiment after a lot of trial and error basis. A powerful global search ability is obtained by this operation.

Step: 4 Crossover:

Here, it comprises of 2 steps:

(1) Initially, a binary integer value matrix map is generated which is of size S*D
2) Secondly, depending on the matrix map generated, the location of crossover individual elements are determined in population *A*
3) Therefore, to get the final trial population *T*_*p*_, the individual elements in *A* are exchanged with the respective collaborating positive elements in population *V*. The expression of crossover operation is expressed as

(36) Ri,j={Ai,j⁢⁢i⁢fm⁢a⁢pi,j=1Vi,j               ⁢⁢⁢o⁢t⁢h⁢e⁢r⁢w⁢i⁢s⁢e

Sometimes they might be an overflow of few individuals of the trial population *T* than the allowed search space limits after the crossover operation. There will be a regeneration of individuals present beyond the boundary control based on equation (32).

Step 5: Selection II phase:

To preserve the best favorable trial individuals, a greedy selection mechanism is utilized. For the trial individuals and the target individuals, the fitness values are compared. The trial individual can get accepted to the next generation if the fitness value of trial individuals is much less than the target individuals. If the fitness merit and utility of trial individuals are more than the target individual, then the target individual is retained in the population. The definition of selection operation is expressed as follows:


(37) $$A_{i}=\left\{ \begin{array}{c} T_{p_{i}},\begin{array}{cc} i\,f & f\,i\,t\,n\,e\,s\,s\,\left(T_{p_{i}}\right)<f\,i\,t\,n\,e\,s\,s\,\left(A_{i}\right) \end{array}\\ A_{i},\begin{array}{cccc} & & & \begin{array}{cccc} & & & o\,t\,h\,e\,r\,w\,i\,s\,e \end{array} \end{array} \end{array}\right.$$

where the objective function value of a particular individual is *f* (⋅).

### Feedback mechanism for swarm computing techniques

To manually label all the sparse feature representations, it is pretty time-consuming and very expensive too. Therefore, a feedback technique is introduced that can automatically deal and relate whether an unlabeled sparse representation is chosen and present in a training data pool once the HMM assigns it with the signal feature. Therefore, the best strategy is to calculate the entropy measures of a sparse representation *S* so that the signal is more discriminating than all the other signal features on the sparse representations *S*. A famous information theoretic measure it is expressed as


(38) $$\phi \,\left(S\right)=-\sum_{i}P\,\left(f_{i}|S\right)\,\log P\,\left(f_{i}|S\right)$$

where *P* (*f*_*i*_|*S*) expresses the probability of the sparse representations *S* recognized as a signal feature *f*_*i*_. If ϕ(*s*) is less, then the certainty about the sparse representations *S* on the signal feature *f*_*i*_ is more. To decide whether a sparse representation should be present in the training data set, the algorithm of feedback-based mechanism is utilized as shown in Algorithm 3.

**Algorithm 3:** Feed back mechanism.

Input: training data *D*, test pool data *N*, query strategy parameter *ϕ*(•), query batch size parameter *B*_*s*_ Repeat For i=1 to |*F*| do Optimized λ^(*f*_*i*_)^ by utilizing current *D* and PSO/DE/WOA/BSA algorithm End for For *b*_*s*_ = 1 *to B*_*s*_ *do* $S_{b_{s}}^{{}^{*}}=\underset{S\in U}{\arg\max}\phi \,\left(S\right)$ Move $S_{b\,s}^{{}^{*}}$ from *N* to *D* End for Utilizing some stopping criterion

The gist of EEG signal classification with sparse representation measures and a swarm computing-based HMM methodology is as follows:

(a) Preprocessing of signals is done initially by using Independent Component Analysis (ICA).
(b) Sparse Modeling of the signals is done.
(c) Computation and extraction of sparse feature vectors of the entire dataset are done.
(d) Building an HMM for the assessed sparse signal features as observed variables.
(e) The hidden states of each λ^(*f*_*i*_)^ are optimized by PSO/DE/WOA/BSA.
(f) For every λ^(*f*_*i*_)^ (*f*_*i*_ ∈ |*F*|), the signal vector [*w*_1,_*w*_2_, …, *w*_*n*_]^(*f*_*i*_)^ of *S* in sparse signal feature *f*_*i*_ is computed, and the output values *P* ([*w*_1_, *w*_2_, …, *w*_*n*_]^(*f*_*i*_)^|λ^(*f*_*i*_)^) are calculated through model λ^(*f*_*i*_)^.
(g) Return f* = arg max_*f*_*i*_∈|*F*|_ {*P* ([*w*_1_, *w*_2_, …, *w*_*n*_]|λ^(*f*_*i*_^)}.

To test the performance of every HMM, several sparse representation features represented as observed variables are selected randomly. For each signal representation, the test dataset contains numerous sparse representation features. For about ten times, each test result is executed, and the evaluation is based on the average results.

## Deep learning–based methodology

**FIGURE 2:**
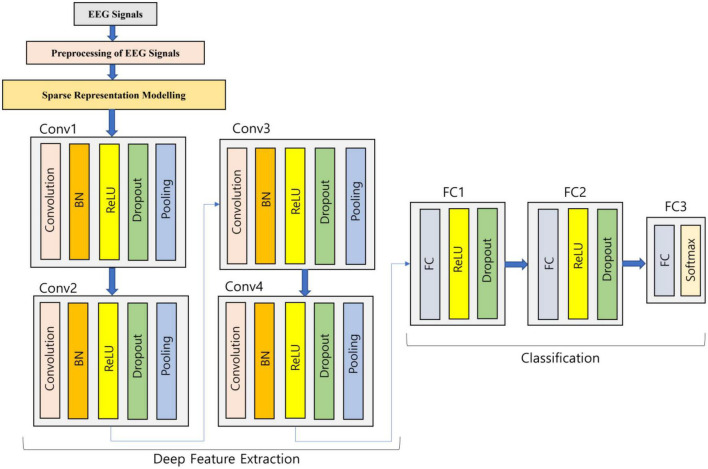
Deep learning 1D-CNN for the classification of EEG.

Generally, to perform the classification in an end-to-end manner, the deep CNN model (Zhao et al., 2020) is utilized but in this work, once the sparse representation modeling to EEG signals is done, then deep feature extraction happens through the developed deep learning model, and finally, it is fed to classification. The utilized 1D-CNN deep learning architecture is expressed in Figure 2 as follows:

The sparse represented EEG signals are fed into the four convolution blocks where every block is comprised of five different layers so that the sparse representation can be learned more deeply. For the generation of a group of linear activation responses, multiple convolutions in parallel are computed by the first layer. In order to solve the internal variable shift, the second layer utilized is Batch Normalization (BN). A nonlinear activation function in the layer is passed by each linear activation response. Rectified Linear Unit (ReLU) is the chosen activation function and is implemented in this work. To avoid overfitting, the concept of dropout methodology is used in the fourth layer. Finally, translation invariance is introduced by the max pooling layer, which serves as the last layer in the block. In the developed deep learning architecture, the second, third, and fourth convolution blocks are same as the first convolution block repeating the same actions. The flattening of the feature maps is done into a one-dimensional vector at the end of the fourth convolution block which is connected to the Fully Connected (FC) layer so that the features are integrated. The activation function is chosen as ReLU for the first two FC layers which are accompanied by a dropout layer. Softmax activation function is implemented in the third FC layer so that a vector of probabilities communicating to every category is given as output. The experiments were tried with various model parameters and the one which produced good results is provided in this work.

### Convolution layer

In order to process the data with same network structures, CNN is widely preferred. By means of regular sampling on time axis, the consideration of the time series data can be done as a one-dimensional grid. The important three layers, namely, convolution layer, activation function layer, and pooling layer are present in any convolutional block of the standard CNN model. The convolution operation for the 1D EEG data utilized in this article is expressed as:


(39) $$s\,\left(t\right)=\left(x^{*}\,w\right)\,\left(t\right)=\sum_{a}x\,\left(a\right)\,w\,\left(t-a\right)$$

The attributes of the sparse interaction are present in the convolutional network that helps to mitigate the storage requirements of the developed deep learning model. This ensures that all the memory parameters are thoroughly learned with the parameters shared by the convolution kernel. Convolution is actually a special type of linear operation and it is only with the help of activation function, the nonlinear characteristics are bought in the network. The commonly utilized activation function in CNN is ReLU, which helps to solve the vanishing gradient issue so that the models can learn faster and enhance the overall performance. The spatial size of the representation is mitigated with the help of pooling function so that the total number of parameters along with the computation is reduced in the network. At specific portions, the output of the system is replaced by the pooling function, thereby making the representation roughly invariant to minor input translations.

### Computation of batch normalization

To the standard convolution blocks, the addition of the BN layer along with the dropout layer is done. There is always a close relation between the parameters of every layer where the training of the deep neural network is done. When the input layers are distributed, an inconsistency occurs causing an issue called as internal covariate shifts, making it hectic to choose a suitable learning rate. Therefore, BN process is used in this study so that almost any deep network can be reparametrized quite easily by means of coordinating the updation process between multiple layers of the network. Therefore, the normalization is considered as part of the deep learning model architecture and it helps to normalize every mini-batch. For the mini-batch response *H*, the computation of the sample mean (μ) and standard deviation (σ) in backpropagation during training is done as follows:


(40) $$\mu =\frac{1}{m}\,\sum_{i}H_{i}$$

(41) $$\sigma =\sqrt{\delta +\frac{1}{m}\,\sum_{i}{\left(H-\mu \right)}_{i}^{2}}$$

To prevent the gradient from becoming undefined, the delta component δ is usually added and it is a very small positive value. In order to normalize *H*, the following expression is utilized as:


(42) $$H^{\prime }=\frac{H-\mu }{\sigma }$$

The convergence of the training phase can be well accelerated by BN so that overfitting can be avoided easily and therefore BN is employed after every convolution layer.

### Fusion of features along with classification

A large number of parameters need to be learned by the deep neural networks and in the case of smaller datasets, there is a high chance for occurrence of overfitting. Therefore, to solve this issue, dropout technology was added so that the coadaptation of feature detection is avoided fully. The random dropping of units with a predefined probability from the neural network seems to be the main intention of dropout layer during the training process. When compared to other regularization methods, this technique can reduce the overfitting to a great extent and therefore after each ReLU activation function, a dropout layer is added. The high-level features of the EEG signals are indicated by the output of the final convolutional block. The FC layer can easily learn all the nonlinear combinations of these functions. In this work, three FC layers have been developed. The connection of all the neurons in the last max-pooling layer is done with the neurons of the first FC layer. Depending on the final classification problem, the determination of the total number of neurons in the final FC layer is done and since a two-class epilepsy classification problem and a two-class schizophrenia classification problem is dealt in this study, the number of neurons in FC3 layer is chosen to be two. A generalized form of the binary manifestation of logistic regression is the softmax activation function. In order to assemble a categorical distribution over the class labels and to trace the probability of every input element belonging to a particular label, this softmax function is usually implemented in the ultimate layer of a deep neural network. The respective probability of the *i^th^* sample expressed by *x*^(*i*)^ which belongs to each category and is indicated by the softmax function *h*_θ_(*x*^(*i*)^) as follows:


(43) $$h_{\theta }\,\left(x^{\left(i\right)}\right)=\left[\begin{array}{c} p\left(y^{\left(i\right)}=1|x^{\left(i\right)};\theta \right)\\ p\left(y^{\left(i\right)}=2|x^{\left(i\right)};\theta \right)\\ :\\ p(y^{\left(i\right)}=k|x^{\left(i\right)};\theta \end{array}\right]=\frac{1}{\sum_{l=1}^{k}e^{\theta_{l}^{T}\,x^{\left(i\right)}}}\,\left[\begin{array}{c} e^{\theta_{1}^{T}\,x^{\left(i\right)}}\\ e^{\theta_{2}^{T}\,x^{\left(i\right)}}\\ :\\ e^{\theta_{k}^{T}\,x^{\left(i\right)}} \end{array}\right]$$

where the softmax model parameters are expressed by *θ*_1_, *θ*_2_, …, *θ*_*k*_.

### Model training

The weight parameters are required to be learned from the EEG data for the training of the proposed model. The standard Backpropagation algorithm was used and the loss function utilized is cross entropy. The stochastic gradient descent technique with Adam optimization is utilized to learn the parameters. The hyperparameters of Adam are set as follows: learning rate is 0.0001, beta1 value is set at 0.5 and beta2 value is set at 0.55. The batch size is considered as 200 in our experiment which helps in the updation of the training process. The total number of epochs utilized in this work is expressed as 250 so that the training of the model can be done well.

## Results and discussion

For evaluating and validating this proposed model, it has been tested on University of Bonn dataset (Andrzejak et al., 2001) which deals with epilepsy classification and the schizophrenia dataset from Institute of Psychiatry and Neurology in Warsaw, Poland, which deals with schizophrenia classification (Olejarczyk and Jernajczyk, 2017). There are five sets of epileptic data available such as A, B, C, D, and E. Set A and B belongs to the normal category, Set C and D belongs to the inter-ictal category, and set E belongs to the ictal category. The classification problem considered in epileptic dataset are A-E, AC-E, B-E, CD-E, ACD-E, and ABCD-E, and the classification problem considered in schizophrenia datasets are normal versus schizophrenia. The elaborate details of both datasets are given in Andrzejak et al. (2001) and Olejarczyk and Jernajczyk (2017). For both datasets, the Independent Component Analysis (ICA) is utilized as a common pre-processing technique. As far as the epilepsy dataset is considered, 100 single-channel recordings of EEG signals are present in each of these sets with a sampling rate of 173.61 Hz and time duration of 23.6 s. The respective time series is sampled into 4097 data points and further every 4097 data point is divided into 23 chunks, thereby the total number in each category has about 2,300 samples. For deep learning methodology, once the sparse modeling is implemented to it, the random division of the 2,300 EEG samples is done into ten non-overlapping folds as a 10-fold cross-validation is adopted here for evaluation. As far as the SI-based HMM along with the conventional machine learning is considered, the 2,300 samples are reduced by means of sparse feature extraction eliminating the redundant ones. Only the essential sparse features are considered as observed variables as expressed in the sparse representation modeling concept and then it is proceeded for classification by the SI-based HMM and the conventional machine learning models. As far as the schizophrenia dataset is concerned, there are about 225,000 samples with each channel, and the data are represented in this study with a matrix of [5,000 × 45]. As there are 19 such channels available there, it is represented as [5,000 × 45 × 19]. For the deep learning methodology, once the sparse modeling is implemented to it, the random division of the schizophrenia EEG samples is done into ten non-overlapping folds as a 10-fold cross-validation is adopted in this study for evaluation. As far as the SI-based HMM along with the conventional machine learning is considered for schizophrenia EEG signal classification, the [5,000 × 45] data are reduced by means of sparse feature extraction eliminating the redundant ones. Only the essential sparse features are considered as observed variables as represented in the sparse representation modeling concept and then it is proceeded for classification by the SI-based HMM and the conventional machine learning models. The performance metrics analyzed are the general measures used widely such as Classification accuracy, Sensitivity, and Specificity. The details of the 1D-CNN model utilized in this research are tabulated in Table 2.

**TABLE 2:** Convolutional neural network (CNN) structure details utilized in this work.

Name of the block	Types of layer	Number of neurons	Kernel size (output feature map)	Stride
Conv1	Convolution	179 × 20	60	1
	BN	179 × 20	–	–
	ReLU	179 × 20	–	–
	Dropout	179 × 20	–	–
	Max-pooling	90 × 20	2	2
Conv2	Convolution	71 × 40	40	1
	BN	71 × 40	–	–
	ReLU	71 × 40	–	–
	Dropout	71 × 40	–	–
	Max-pooling	36 × 40	2	2
Conv3	Convolution	31 × 60	20	1
	BN	31 × 60	–	–
	ReLU	31 × 60	–	–
	Dropout	31 × 60	–	–
	Max-pooling	18 × 60	2	2
Conv4	Convolution	13 × 80	10	1
	BN	13 × 80	–	–
	ReLU	13 × 80	–	–
	Dropout	13 × 80	–	–
	Max-pooling	5 × 80	2	2
FC1	FC	64	–	–
	ReLU	64	–	–
	Dropout	64	–	–
FC2	FC	32	–	–
	ReLU	32	–	–
	Dropout	32	–	–
FC3	FC	2	–	–

Table 3 indicates the performance analysis of the proposed SI-based HMM for different datasets with optimization techniques. The highest sensitivity of 100% is attained for Schizophrenia dataset with DE-HMM, WOA-HMM, and BSA-HMM methods. In the case of epileptic dataset (AC-E) with BSA-HMM, and epileptic dataset (B-E) with WOA-HMM also, it reached 100% sensitivity. The lower sensitivity value of 69.86% is reached for epileptic dataset (AC-E) with WOA-HMM method. The highest specificity of 100% is obtained for epileptic dataset (AC-E) with PSO, DE, and WOA-based HMM methods. As in the case of epileptic dataset (A-E) with DE-HMM and epileptic dataset (B-E) with DE and BSA-based HMM methods, it reached 100% specificity. A low specificity value of 76.83% is reached for schizophrenia dataset with DE-HMM method. A high classification accuracy of 95.70% is attained for epileptic dataset (A-E) with DE-HMM method and low classification accuracy of 82.43% is reached for epileptic dataset (ABCD-E) with BSA-HMM method. For schizophrenia datasets, a high classification accuracy of 91.41% is obtained with PSO-HMM, and a low classification accuracy of 88.41% is obtained from DE-HMM.

**TABLE 3:** Performance analysis of the proposed swarm intelligence based HMM for different datasets.

Performance metrics (%)	Datasets	Swarm intelligence based HMM			
		PSO-HMM	DE-HMM	WOA-HMM	BSA-HMM
Sensitivity	Epileptic dataset (A-E)	93.26936	91.40875	83.12805	95.83567
	Epileptic dataset (AC-E)	79.03875	89.84875	69.86516	100
	Epileptic dataset (B-E)	94.53375	90.88625	100	85.69125
	Epileptic dataset (CD-E)	88.38541	89.83978	83.87825	84.27894
	Epileptic dataset (ACD-E)	85.34346	88.38376	82.47892	82.47892
	Epileptic dataset (ABCD-E)	87.46243	85.38761	81.17835	81.48923
	Schizophrenia dataset	92.97457	100	100	100
Specificity	Epileptic dataset (A-E)	90.36875	100	86.52344	90.1125
	Epileptic dataset (AC-E)	100	100	100	83.4325
	Epileptic dataset (B-E)	90.36875	100	77.02032	100
	Epileptic dataset (CD-E)	89.19385	91.46782	87.47892	85.56672
	Epileptic dataset (ACD-E)	86.37892	89.23872	86.37892	86.38997
	Epileptic dataset (ABCD-E)	90.34678	88.22389	83.35781	84.37781
	Schizophrenia dataset	89.85625	76.8375	79.81938	81.27457
Classification accuracy	Epileptic dataset (A-E)	91.81906	95.70435	84.82575	92.97409
	Epileptic dataset (AC-E)	89.51938	94.92435	84.93258	91.71625
	Epileptic dataset (B-E)	92.45125	95.44312	88.51016	92.84563
	Epileptic dataset (CD-E)	88.78963	90.65381	85.67858	84.92283
	Epileptic dataset (ACD-E)	85.86119	88.81124	84.42892	84.43445
	Epileptic dataset (ABCD-E)	88.90460	86.80575	82.87462	82.434445
	Schizophrenia dataset	91.41541	88.41875	89.90969	90.63729

Table 4 shows the performance analysis of the proposed methodology for the biosignal processing datasets in terms of accuracy using swarm intelligence–based HMM, conventional machine learning, and deep learning techniques. If the proposed flow of methodology is implemented with NBC for the datasets, then a high classification accuracy of 92.12% is obtained for the B-E dataset. When the standard LDA is utilized, then a high classification accuracy of 92.34% is obtained for the A-E dataset, and low classification accuracy of 80.5% is obtained for the ACD-E dataset. When KNN methodology is utilized, a high classification accuracy of 90.23% is obtained for A-E dataset and a low classification accuracy of 79.98% is obtained for ABCD-E dataset. If the proposed flow of methodology is implemented with Adaboost classifier for the datasets, then a high classification accuracy of 89.34% is obtained for the B-E dataset. When comparing all the conventional classifiers, the SVM performs better as a higher classification accuracy of 93.49% is obtained for the schizophrenia dataset and low classification accuracy of 87.9% is obtained ABCD-E dataset. Before computing the swarm intelligence–based HMM model, the methodology was tested for the ordinary HMM model and the highest result of only 87.34% was obtained for the A-E dataset. This seemed to motivate the researchers to undergo more research in fine-tuning HMM so that a better result could be obtained. The swarm techniques were successfully computed with HMM, and much better results were obtained. For the PSO-HMM combination, a higher classification accuracy of 92.45% was obtained for the B-E combination and a lower classification accuracy of 85.86% was obtained for the ACD-E combination. For the DE-HMM combination, a higher classification accuracy of 95.7% was obtained for the A-E combination and a lower classification accuracy of 86.8% was obtained for the ABCD-E combination. For the WOA-HMM combination, a higher classification accuracy of 89.9% was obtained for the schizophrenia dataset and a lower classification accuracy of 82.87% was obtained for the ABCD-E combination. For the BSA-HMM combination, a higher classification accuracy of 92.97% was obtained for the A-E dataset and a lower classification accuracy of 82.43% was obtained for ABCD-E combination. For the proposed 1D-CNN combination, a higher classification accuracy of 98.94% was obtained for the A-E dataset, and a lower classification accuracy of 97.05% was obtained for ACD-E combination.

**TABLE 4:** Performance analysis of sparse representation based swarm HMM and deep learning for the biosignal processing datasets in terms of accuracy.

Classifier	A-E	AC-E	B-E	CD-E	ACD-E	ABCD-E	Schizophrenia
NBC	91.37582	87.34981	92.12783	85.01358	82.10368	81.89451	87.03481
LDA	92.34589	86.24951	91.34591	86.93169	80.50275	83.67912	85.56921
KNN	90.23578	85.34917	89.25791	85.87615	81.28507	79.98205	88.45917
Adaboost	88.98659	83.45691	89.34725	87.58941	77.28905	75.91632	86.68113
SVM	93.45781	91.87543	92.46915	91.34721	88.56891	87.90982	93.49812
HMM	87.34591	81.12678	83.45916	84.33861	79.48697	71.26748	81.36991
**PSO-HMM**	91.81906	89.51938	92.45125	88.78963	85.86119	88.90460	91.41541
**DE- HMM**	95.70435	94.92435	95.44312	90.65381	88.81124	86.80575	88.41875
**WOA-HMM**	84.82575	84.93258	88.51016	85.67858	84.42892	82.87462	89.90969
**BSA-HMM**	92.97409	91.71625	92.84563	84.92283	84.43445	82.43444	90.63729
**1D-CNN**	98.94919	97.15912	98.56781	97.56789	97.05981	97.34862	98.19864

### Comparison of results with previous works associated with similar datasets

The authors in recent years have dealt with classification problems as per their wish depending on their problem requirement, and therefore, it was not mandatory to perform the analysis of classification on every available subset of the epileptic data. Therefore, the available results are compared with our works and projected in Table 5.

**TABLE 5:** Performance comparison of our works with the previous works – Epilepsy dataset.

References	A-E	AC-E	B-E	CD-E	ACD-E	ABCD-E
Bhardwaj et al., 2016	98.64	–	–	–	98.61	98.89
Peker et al., 2016	99.50	–	–	–	–	99.13
Riaz et al., 2016	99.00	–	–	–	–	96.00
Diykh et al., 2017	100	–	99.76	–	96.50	94.00
Zhang and Chen, 2017	–	–	–	–	–	98.87
Raghu et al., 2017	99.70	–	–	–	–	–
Ullah et al., 2018	100	–	99.6	99.7	–	99.7
Li et al., 2018	–	–	–	91.00	–	–
Sharma et al., 2018	100	–	–	–	–	–
Raghu et al., 2019	99.45	96.50	96.06	96.85	96.00	97.20
Gupta and Ram, 2019	99.50	–	99.50	99.00	–	98.60
Turk and Ozerdem, 2019	99.50	–	99.50	–	–	–
Sameer and Gupta, 2020	98	–	96	96.33	–	97.40
Zhao et al., 2020	99.52	–	99.11	98.03	–	98.76
**Proposed technique 1:** Sparse representation measures with PSO-HMM [2022]	91.81	89.51	92.45	88.78	85.86	88.90
**Proposed technique 2:** Sparse representation measures with DE-HMM [2022]	95.70	94.92	95.44	90.65	88.81	86.80
**Proposed technique 3:** Sparse representation measures with WOA-HMM [2022]	84.82	84.93	88.51	85.67	84.42	82.87
**Proposed technique 4:** Sparse representation measures with BSA-HMM [2022]	92.97	91.71	92.84	84.92	84.43	82.43
**Proposed technique 5:** Sparse representation measures with 1D-CNN [2022]	**98.94**	**97.15**	**98.56**	**97.56**	**97.05**	**97.34**

On analyzing Table 5, it is quite evident that a wonderful attempt has been made by the authors to attain good classification accuracy results. As far as the A-E epileptic dataset is considered, among the proposed methodology, the sparse representation measures with 1D-CNN surpassed all the other results proposed in this work and gave the highest classification accuracy of 98.94% for A-E dataset, 97.15% for AC-E dataset, 98.56% for B-E dataset, 97.56% for CD-E dataset, 97.05% for ACD-E dataset, and 97.34% for ABCD-E dataset. When the swarm intelligence–based HMM is concerned, the highest classification accuracy of 95.70% is obtained when the sparse representation measures are implemented with DE-HMM for the A-E dataset. Similarly, the DE-HMM gives a high classification accuracy of 94.92% in AC-E dataset, 95.44% in B-E dataset, 90.65% in CD-E dataset, and 88.81% in ACD-E dataset when compared to other swarm-based HMM methods. For the ABCD-E dataset, the sparse representation measures with PSO-HMM provided a high accuracy of 88.9% when compared to other swarm-based methods. It is commonly known that deep learning outperforms most of the conventional pattern recognition techniques and so in this work also, the highest classification accuracy of 98.94% is obtained with the novel idea of sparse modeling with deep learning. When the results of the present work are compared to the previous works, the deep learning results obtained by us have matched more or less similar to the results obtained by the previous methods though at many places, the classification accuracy obtained by this work is slightly lower than the earlier proposed works by a range of two to four percent. In such a case, it should not induce the research community into thinking that as the classification results are slightly lower, the proposed methodology is not as versatile as the other methods. It has to be observed and noted that in the field of machine learning, the classification accuracies may be more or less in the range of plus or minus 3–5%, but what has to be observed carefully is the ease of methodology and implementation strategy. If that aspect is considered, the proposed methodology surpasses many earlier techniques as no strong mathematical model has been built in earlier models, whereas a strong mathematical model for sparse representation with the hybrid SI-based HMM along with deep learning is done in this work. Moreover, swarm intelligence field is like an ocean and there are hundreds of algorithms developed in the past two decades by various researchers. This work is just a starting step to use the concept of sparse modeling with SI-based HMM. In the upcoming years, a variety of other SI algorithms shall be implemented to HMM to test its ability and check its performance with the sparse representation models, and the authors are confident of obtaining much higher classification accuracy. As far as the schizophrenia classification analysis is concerned, very high-quality literature is not available online as it is an emerging field. All the important works in schizophrenia classification are discussed in the introduction section of the article with their respective classification accuracies, where reference (Prabhakar et al., 2020a) reported 98.77%, reference (Prabhakar et al., 2020b) reported 92.17%, and reference (Oh et al., 2019) reported 81.26% for subject-based testing and 98.51% for non-subject-based testing. However, when comparing our results with the previous works, the concept of sparse representation with 1D-CNN produced a very high classification accuracy of 98.19%, and the concept of sparse representation with SI-based HMM produced an accuracy of 91.41% for PSO-HMM, 88.41% for DE-HMM, 89.90% for WOA-HMM, and 90.9% for BSA-HMM. Every swarm intelligence technique is so inspiring and it would take a life time to understand why a particular combination with sparse representation measures performs better with HMM or deep learning. Possible ways to obtain better results in SI-based HMM is to fine-tune the parameters much more carefully, varying the hyperparameters depending on the problem requirement, increasing the iteration numbers if the pre-requisite conditions are not satisfied, enhancing the essential parameters of the algorithm depending on the SI techniques considered, and updating the state space model of the HMM effectively by efficient techniques. Better results could also be obtained by means of utilizing other hybrid deep learning methods for the efficient classification of biomedical signals. An interesting classification tool based on fuzzy similarities which are characterized by a low computational complexity, and high utility for real-time applications is proposed in Versaci et al. (2022). Although it was tested on a NdT problem, due to the transversality of the approach, the method could be easily applied to the problem studied in this work too, and the authors wanted to implement a similar strategy utilized in Versaci et al. (2022) to the analysis of neurological disorders in future.

## Conclusion and future work

An efficient modality through which brain signals corresponding to different states can be acquired easily is by means of using EEG. In this article, sparse representation and modeling of EEG signals are done initially, and later, an HMM classification model was proposed to compute the hidden states in the HMM, four different types of SI techniques were incorporated to make the HMM very flexible. This kind of methodology involving sparse representation with a pliable HMM for biosignal classification seems to be very efficient and easy to handle. An exhaustive analysis of the proposed SI-based HMM for epileptic and schizophrenia datasets was computed and comprehensively analyzed. The sparse representation modeling was also combined with deep learning, and conventional machine learning techniques and exhaustive analysis are provided. When the proposed sparse representation measures were combined with SI-based HMM, the highest accuracies reported are 92.45% for PSO-HMM, 95.7% for DE-HMM, 89.9% for WOA-HMM, and 92.97% for BSA-HMM. When the proposed sparse representation measures were utilized with deep learning by utilizing a CNN, high accuracy of 98.94% was obtained. Future works aim to develop more efficient sparse representation models by means of introducing more advanced concepts in the synthesis and analysis side. Though the sparse representation–based swarm HMM methods did not provide very high classification accuracy when compared to other previous works, the careful selection of the swarm intelligence algorithm with HMM would aid a very high classification accuracy with less error rate in the upcoming years. Future works also aim to hybrid the sparse representation measures with other nature-inspired algorithms such as Ant Colony Optimization (ACO), Artificial Bee Colony (ABC), Genetic Bee Colony (GBC), Cuckoo Search Optimization (CSO), Spider Monkey Optimization (SPO), Bat algorithm, and Firefly algorithm, so that the hidden states of the HMM can be well computed in order to assess its performance on the biomedical signal datasets. Other work plans to incorporate in future include the usage of sparse representation measures with efficient deep learning techniques, such as Long Short-Term Memory (LSTM), Bidirectional LSTM, Gated Recurrent Unit (GRU), Bidirectional GRU, and hybrid deep learning techniques, for efficient classification of epilepsy and schizophrenia from its respective datasets. This proposed kind of methodology is also planned to be implemented in other image processing datasets, stock market datasets, speech processing datasets, and other beneficial datasets to check its performance assessment. In the upcoming years, the work can be integrated with Very Large Scale Integration (VLSI) technology to produce some good advancement in medicine and technology for the betterment of human health care.

## Data availability statement

The raw data supporting the conclusions of this article will be made available by the authors upon reasonable request, without undue reservation.

## Author contributions

All authors listed have made a substantial, direct, and intellectual contribution to the work, and approved it for publication.
